# Epitranscriptomic profiling of m5C RNA methylation reveals a dynamic response to TSWV infection in tomato

**DOI:** 10.3389/fpls.2025.1722290

**Published:** 2026-01-22

**Authors:** Xinyi Zhao, Yanwei Gong, Yubing Jiao, Wanhong Zhang, Dong An, Yingwen Wang, Binna Lv, Ying Li, Lili Shen, Jinguang Yang

**Affiliations:** Key Laboratory of Tobacco Pest Monitoring Controlling & Integrated Management, Tobacco Research Institute of Chinese Academy of Agricultural Sciences, Qingdao, China

**Keywords:** m5C, m5C-RIP-seq, RNA stability, *Solanum lycopersicum*, TSWV

## Abstract

Cytosine-5 methylation (m5C) is a crucial epitranscriptomic mark in eukaryotes that modulates RNA stability and gene expression. While the roles of m5C are partially understood in model plants, its function in horticultural crops under biotic stress remains largely unexplored. To address this gap, we investigated the role of m5C modification in tomato response to tomato spotted wilt virus (TSWV) infection. We constructed the first comprehensive m5C epitranscriptomic map in Solanum lycopersicum. To investigate its role in plant immunity, we further profiled the dynamic changes of the m5C methylome upon TSWV infection, followed by integrative multi-omics analysis. Functional validation was performed through virus-induced gene silencing (VIGS) of the key RNA methyltransferase gene *SlTRM4B*. The m5C epitranscriptomic map revealed conserved modification patterns with enrichment at transcription start and stop sites. Upon TSWV infection, a global increase in m5C modification levels was observed across the transcriptome, which was correlated with the significant upregulation of RNA methyltransferase (RCMT) family genes, particularly *SlTRM4B*. Integrative multi-omics analysis revealed that genes exhibiting both hypermethylation and increased expression were significantly enriched in the plant-pathogen interaction pathway. VIGS of *SlTRM4B* demonstrated that this methyltransferase is essential for maintaining the stability of its target transcripts under TSWV infection, leading to enhanced disease susceptibility. Collectively, our findings demonstrate that *SlTRM4B*-mediated m5C RNA methylation fine-tunes post-transcriptional regulation to reprogram the transcriptome for disease resistance in tomato. This work provides novel insights into the epitranscriptomic mechanisms governing plant responses to viral pathogens.

## 1 Introduction

Tomato spotted wilt virus (TSWV), a member of the species Orthotospovirus within the order Bunyavirales, is a devastating plant pathogen. Its genome consists of three single-stranded RNA segments (L, M, S) that encode an RNA-dependent RNA polymerase, glycoproteins, a movement protein, a nucleocapsid protein, and a silencing suppressor. TSWV is notable for its exceptionally broad host range, capable of infecting over 1,000 plant species, including major economic crops such as tomato, pepper, and tobacco. The virus has a global distribution, causing particularly severe economic losses in temperate and subtropical regions worldwide. This combination of biological characteristics underscores the significant threat TSWV poses to global agriculture and highlights the importance of researching host resistance mechanisms (Qi et al., 2021).

In epitranscriptomics, more than 170 distinct types of RNA chemical modifications have been identified, underscoring their role as post-transcriptional regulatory elements (Lei et al., 2023). These modifications are vital for fine-tuning gene expression, maintaining RNA stability, and ensuring translational fidelity. Therefore, they broaden our understanding of the complex regulatory network governing RNA metabolism (Motorin and Grosjean, 1999; Nachtergaele and He, 2018). Chemical modifications are found in nearly all cellular RNA types, including coding (mRNA) and non-coding RNAs (e.g., tRNA, rRNA, lncRNA, and miRNA), making them pervasive regulatory features at the transcriptome level (Roundtree et al., 2017). Among these modifications, methylation is the most extensively studied category. Prominent examples include N6-methyladenosine (m6A), 5-methylcytosine (m5C), N7-methylguanosine (m7G), pseudouridine (Ψ), and N1-methyladenosine (m1A). Given the widespread implications of these modifications, we focus specifically on 5-methylcytosine (m5C), which involves the methylation of cytosine at its fifth carbon position. This modification was first identified in *E. coli* RNA in 1958 (Amos and Korn, 1958). Subsequently, researchers discovered m5C modification in mouse mRNA. Further studies have demonstrated that this modification is present in both mRNA and rRNA across eukaryotic systems, including humans and yeast. Thus, m5C is conserved across species (Amort et al., 2017; Zou et al., 2024). The deposition of m5C is catalyzed by methyltransferases, known as “writers,” such as the human NSUN2 protein and its yeast ortholog. These enzymes use S-adenosylmethionine (SAM) as the methyl donor (Fang et al., 2023).

The m5C modification performs multiple functions, including stabilizing RNA, enhancing translation efficiency, promoting transcript transport, and participating in stress and immune responses across various cellular and developmental contexts (Lu et al., 2024a). In mRNA, m5C modification enhances translation efficiency by regulating RNA metabolism, particularly RNA export. In tRNA, m5C modifications are primarily located in the variable loop and the anticodon loop, where they play crucial roles in maintaining tRNA stability and translation accuracy. Furthermore, in rRNA, m5C modification regulates ribosome biogenesis and protein translation efficiency by altering ribonucleoprotein conformations (Lu et al., 2024b). Beyond these fundamental roles, m5C modification is essential under various physiological and pathological conditions. In the HBV life cycle, m5C modification promotes viral replication by maintaining RNA stability, whereas deletion of the m5C methyltransferase NSUN2 inhibits HBV expression (Feng et al., 2023; Ding et al., 2024). Similarly, NSUN2-mediated m5C modification significantly suppresses SARS-CoV-2 replication (Wang et al., 2024). Additionally, NSUN2 plays a key role in regulating Epstein-Barr virus-encoded RNA 1 (EBER1) levels. Knockdown of NSUN2 or loss of RNA m5C modification enhances EBER1 stability, leading to increased cellular levels. Moreover, m5C modification in EBER1 is particularly important for viral replication, as its loss affects RNA stability (Henry et al., 2020). In Drosophila C virus (DCV), the RNA m5C methyltransferase DNMT2 interacts with DCV RNA, triggering an innate immune response and exerting antiviral defense (Chen et al., 2021). Therefore, these findings indicate that m5C modification not only regulates RNA stability and function but also participates in stress responses, gene expression regulation, and pathological processes through multiple molecular mechanisms. Consequently, m5C modification represents a valuable potential therapeutic target and biomarker.

Recent m5C-RIP-seq analyses have mapped the distribution of m5C in *Oryza sativa* and *Arabidopsis thaliana*, revealing its critical role in promoting RNA translation efficiency (David et al., 2017; Tang et al., 2020). This modification exerts diverse and crucial functions by regulating mRNA stability and translation efficiency while participating in processes such as plant growth, development, and environmental responses (Lu et al., 2024b). Furthermore, the deep learning model m5C-ideep has identified specific patterns of m5C modification in plant RNA transcribed regions, underscoring its role in biological processes including gene expression regulation, RNA splicing, and maintenance of mRNA stability (Malebary et al., 2024). In this study, we employed m5C-RIP-seq to detect and establish a transcriptome-wide map of m5C-modified genes in tomato, analyzing the distribution pattern of m5C-modified genes in tomato leaves. Through integrated analysis of m5C-RIP-seq and RNA-seq, we investigated the dynamic changes in tomato mRNA methylation modifications under TSWV infection and their impact on mRNA transcription levels. Our results reveal the potential regulatory mechanisms of m5C in response to environmental stress and its influence on gene stability and function.

## 2 Materials and methods

### 2.1 Plant material, growth condition, and mechanical inoculation

Tomato plants at the four- to six-leaf stage were used in this experiment. The plants were grown under a 16-hour light/8-hour dark cycle at 25 °C with a relative humidity of 60%. TSWV isolate used in this study, designated as SDLY2, was originally isolated from symptomatic tomato plants in Shandong Province, China. The complete genome sequences of its three RNA segments have been determined and deposited in the GenBank database under the following accession numbers: L RNA: MN833242 (8,911 bp), M RNA: MN870629 (4,773 bp), and S RNA: MN861978 (2,971 bp). Plants were mechanically inoculated with TSWV-containing sap (treatment) or with 1× PBS buffer (mock control) via rub-inoculation. All plants were maintained under identical growth conditions. Leaf tissues from both the TSWV-stressed and control groups were harvested at 7 days post-treatment for subsequent analysis.

### 2.2 Plasmid constructs

To construct the *35S:SlTRM4B-GFP* recombinant vector, the full-length coding sequence of *SlTRM4B* was amplified from tomato and cloned into the pCAMBIASuper1300-GFP vector using In-Fusion cloning (ClonExpress II One-Step Cloning Kit, Vazyme, China).

### 2.3 Library construction for RNA-seq and m5C-RIP-seq

RNA-seq and m5C-Seq was performed by Cloudseq Biotech Inc. (Shanghai, China). Briefly, mRNA from Poly(A)+ RNA, purified from total RNA using a GenElute mRNAMiniprep Kit (Sigma-Aldrich, Germany), was removed using a Ribo-zero kit (Illumina, USA). m5C-RIP was performed with a GenSeq m5C RIP Kit (GenSeq Inc., China) according to the manufacturer’s instructions. Both m5C-RIP and input samples were used for library generation with a NEBNext Ultra II Directional RNA Library Prep Kit (New England Biolabs, USA). Library quality was assessed using an Agilent 2100 Bioanalyzer (Agilent Technologies, USA), and sequencing was performed on an Illumina HiSeq 4000 platform (Illumina, USA).

### 2.4 Data analysis of sequencing

Sequencing data were processed for quality control (QC) using the software fastp (version 0.23.0). During library preparation, each molecular fragment was randomly tagged with a unique identifier (UID) sequence. After raw data QC, UID processing software (kcUID) was employed to cluster similar reads under the same UID, enabling error correction and removal of PCR duplicates. This ensured more accurate molecular sequence information and expression quantification. The high-quality clean reads were first aligned to the ribosomal RNA (rRNA) database of the target species using Bowtie2 (version 2.3.4) (Langmead and Salzberg, 2012), and rRNA reads were subsequently removed. The remaining reads were then mapped to the reference genome (https://solgenomics.net/ftp/tomato_genome/annotation/ITAG3.2_release/) using HISAT2 (version 2.1.0) (Kim et al., 2015). Peak calling was performed with the exomePeak tool (version 2.16.0) (Meng et al., 2014)to identify enrichment peaks. The genomic distribution of peaks was analyzed to determine their localization in coding sequences (CDS), intergenic regions, or intronic regions, along with functional annotation of the associated genes. Homer was used to extract sequence regions corresponding to peaks, scan for shared motifs, identify target binding motifs, and construct motif logos.

### 2.5 m5C-RIP-qPCR

For m5C-RIP, 500 μg of total RNA was incubated with an anti-m^5^C antibody (Abcam, UK) in 500 μL of IP buffer (150 mM NaCl, 0.1% NP-40, 10 mM Tris-HCl, pH 7.4) at 4 °C for 4 hours, followed by incubation with 30 μL of anti-mouse antibody-conjugated magnetic beads (NEB, USA) at 4 °C for 2 hours. The beads were then washed six times with 1 mL of IP buffer. The RNA was extracted using TRIzol reagent and quantified by qRT-PCR.

qRT-PCR was performed using the 2×ChamQUniversal SYBR qPCR Master Mix (Vazyme, China), on an Applied Biosystems 7500 Fast Real-Time PCR System. The relative expression levels of target genes were calculated using the 2-ΔΔCt method, while the modification levels of transcripts were assessed using the % Input method. The % Input was calculated using the formula:


$$\%Input=\frac{2(CtInput-CtRIP)}{dilution\;factor}\times 100$$

CtInput and CtRIP represent the Ct values of the Input and IP or IgG samples, the dilution factor is 10. All reactions were performed in triplicate, and results are expressed as mean ± standard deviation (SD) from three independent biological replicates. All primers used for qRT-PCR are listed in **Supplementary Table S1**.

### 2.6 mRNA decay analysis

Tomato leaves were treated with 30 mg/mL actinomycin D (SigmaAldrich, Germany). Total RNA was extracted using TRIzol from 200 mg of leaf tissue at 0, 8 and 24 hours post-treatment. cDNA was synthesized via reverse transcription and used to assess the stability of candidate genes.

### 2.7 RNA extraction and RNA dot blot

For RNA extraction, a total of 200 mg of treated leaves were flash-frozen in liquid nitrogen, ground using a grinder, and then extracted with TRIzol reagent (Vazyme, China). The concentration and purity of RNA were assessed using a NanoDrop 2000 by measuring absorbance at 260 nm and 280 nm. Reverse transcription was performed using TransScript^®^ All-in-One First-Strand cDNA Synthesis SuperMix (Vazyme, China).

The RNA was verified for m5C modification using a dot blot assay. The RNA was heated at 90 °C for 3 minutes, and 1 µL of each sample or dilution was spotted onto a positively charged nylon membrane. The membrane was air-dried and cross-linked using a UV cross-linker at 120 mJ/cm² for 30s to crosslinking the RNA. The membrane was blocked with 5% bovine serum albumin (BSA) in TBST at room temperature for 1 hour, and then incubated overnight at 4 °C with an anti-m5C antibody (Abcam, UK).After washing three times with 1× TBST, the membrane was incubated for 1 hour with a horseradish peroxidase (HRP)-conjugated anti-mouse secondary antibody (1:5000 dilution).Chemiluminescent detection was performed using Immobilon Western Chemiluminescent HRP Substrate, fluorescence signals were visualized by a GelCap image system (Bio-Rad, USA).

### 2.8 Investigating potential m5C modifications in the TSWV genome

Genome-wide identification of m5C modification sites on TSWV RNA was performed using m5C-RIP-seq technology. A brief outline of the procedure is as follows: total RNA was extracted from TSWV-infected tomato plants, fragmented, and subjected to immunoprecipitation using an anti-m5C antibody to enrich methylated RNA fragments. Sequencing libraries were constructed for both the IP sample and the Input control, followed by sequencing on an Illumina platform. The sequencing data were aligned to the TSWV reference genome using exomePeak software, and significant m5C enrichment peaks (parameters: q-value < 0.05) were identified. These peaks represent high-confidence m5C modification sites.

## 3 Results

### 3.1 TSWV infection induces dynamic changes in m5C modification in tomato

**Figure 1:**
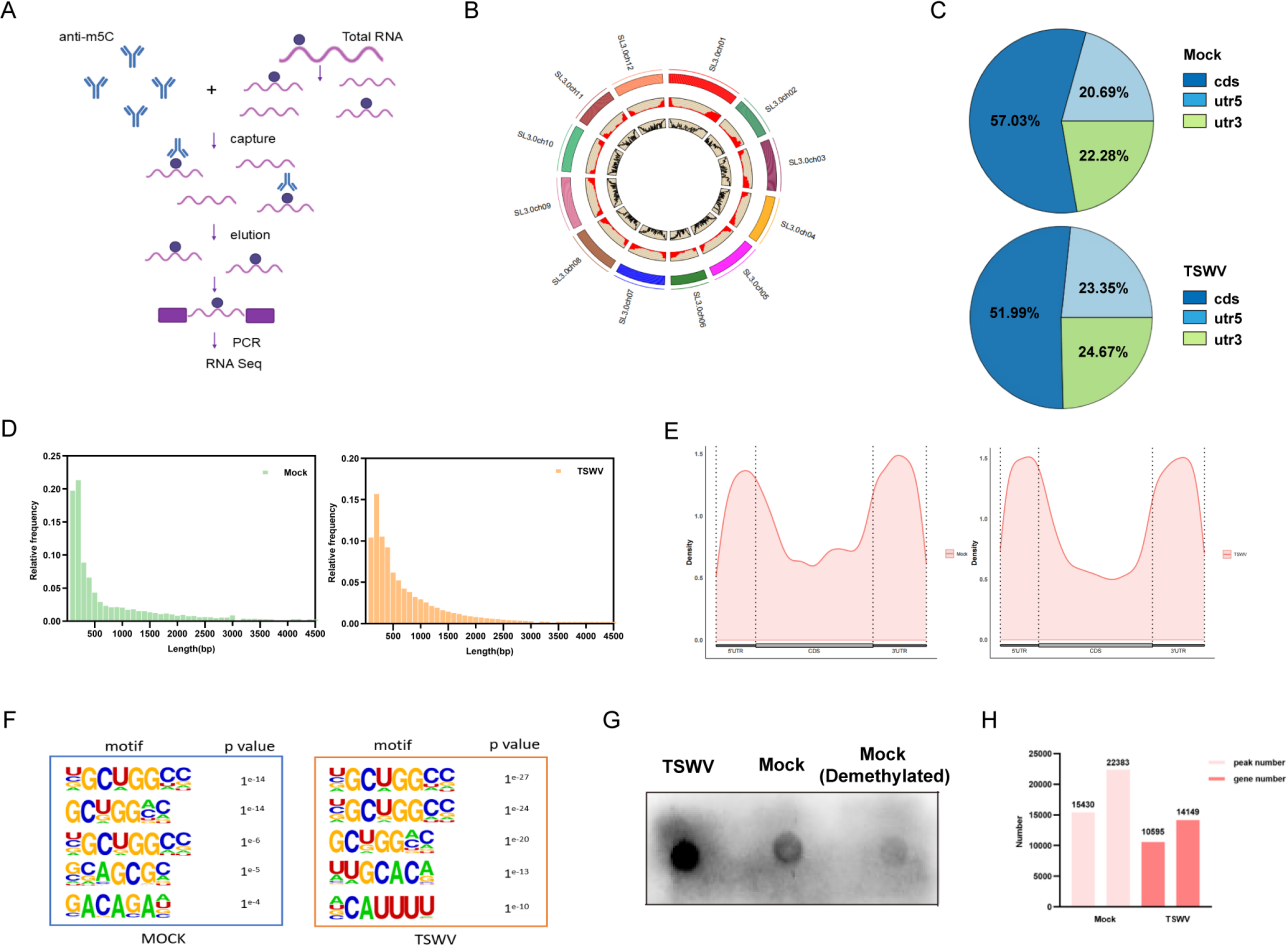
TSWV infection triggers dynamic changes in m5C modification in tomato. **(A)** Schematic workflow for identifying m5C-modified mRNAs in tomato using m5C-RIP- seq technology. **(B)** Circos plot visualizing m5C modification patterns across the tomato genome. From the outside to the inside, the three rings represent: different chromosomes of tomato (1st), the distribution density of m5C modifications across the genome (2nd), and the enrichment level of m5C modifications on chromosome segments (3rd). **(C)** Proportional distribution of m5C-modified transcripts across different functional regions of genes in tomato before and after TSWV infection. **(D)** Length distribution of m5C peaks in target genes before and after TSWV infection. **(E)** Distribution of m5C peaks in tomato transcripts before and after TSWV infection. **(F)** Top five conserved motifs identified within m5C peaks before and after TSWV infection. **(G)** Dot blot validation of global m5C increase upon TSWV infection. **(H)** Number of m5C peaks and associated genes in tomato transcripts under mock and TSWV treatments.

To facilitate a broader analysis of these modifications, we also established an antibody-based m5C-RNA immunoprecipitation and sequencing (m5C-RIP-seq) workflow to generate a transcriptome-wide m5C map in tomato (**Figure 1A**). To investigate the dynamics of m5C modification in tomato following TSWV infection, we performed transcriptome-wide m5C-RIP-seq. Three biological replicates were conducted for each tomato sample to ensure experimental reliability. Following rigorous quality control of the raw sequencing data and removal of low-quality sequences, the proportion of high-quality data exceeded 90%. Each sample generated between 7 million and 20 million genome-aligned sequences, with alignment rates exceeding 90% for all samples, thereby validating the accuracy of the m5C-RIP-seq technique in detecting m5C modifications in tomato mRNA. The genome-wide distribution of m5C modifications in tomato is presented using a circular diagram (**Figure 1B**). The three rings, from outside to inside, display the distribution density of m5C peaks on chromosomes and the enrichment level of m5C modifications on chromosome segments, respectively, comprehensively revealing the genome-wide landscape of m5C modifications in tomato. A total of 15,430 m5C peaks were identified across the tomato genome. These peaks exhibited a specific distribution pattern, predominantly localized within the 50–500 bp interval. Gene annotation results revealed significant enrichment of m5C modifications near the start codon of genes, a finding consistent with observations in humans, Arabidopsis thaliana, and Oryza sativa.

Furthermore, to clarify the distribution preference of m5C modifications within gene regions, we analyzed the proportional distribution of m5C-modified transcripts across different functional regions of genes before and after TSWV infection (**Figure 1C**). Subsequently, we analyzed the distribution characteristics of m5C peaks in tomato samples before and after TSWV infection (**Figure 1D**). The results demonstrated that TSWV infection significantly altered the distribution pattern of m5C peaks in tomato. Compared with the mock group, the morphology of the m5C peaks changed markedly, suggesting that viral infection may remodel the genomic landscape of m5C modification in tomato. Furthermore, the frequency distribution of m5C peak lengths changed following TSWV infection compared to the mock group, indicating that viral infection affects the length characteristics of m5C-modified regions in tomato (**Figure 1E**). The results showed differences in the proportion of m5C modifications across various gene functional regions between the infected and mock groups, suggesting that viral infection may regulate the selective distribution of m5C modifications within gene functional domains. Consensus motif visualization analysis within the m5C peaks was performed using HOMER software (**Figure 1F**). The results indicate that specific conserved motifs were present both before and after TSWV infection, and the top five most enriched motifs changed following infection. The two primary motifs in the mock group were YGCUGGCC and GCUGGMC, while the two primary motifs in the infected group were YCUCUGGCC and CUCUGGCC. These motifs share structural similarity with the CXX sequence found in human mRNA. Similar motifs have also been identified in *Arabidopsis thaliana* and *Oryza sativa*, such as CUUCCU in *Arabidopsis thaliana* and CUYCUCYU in *Oryza sativa*, suggesting that m5C modification motifs exhibit a degree of conservation across different species.

To independently validate the global increase in m5C levels suggested by our sequencing data, we performed an RNA dot blot assay. As shown in **Figure 1G**, a markedly stronger signal was detected in the TSWV-infected sample (spot 1) compared to the mock control (spot 2). Critically, the signal was nearly abolished in the *in vitro* demethylated mock RNA (spot 3), confirming the specificity of the antibody for m5C. Together, these results provide direct biochemical evidence that TSWV infection leads to a substantial increase in global RNA m5C levels. Finally, we quantified the number of m5C peaks and associated genes in tomato samples under mock and TSWV treatments (**Figure 1H**). In the mock group, there were 15,430 m5C peaks associated with 10,595 genes; following TSWV infection, the number of m5C peaks increased to 22,383, and the number of associated genes rose to 14,149. These results indicate that TSWV infection significantly upregulates the global level of m5C modification in the tomato transcriptome, as evidenced by the substantial increase in both the number of m5C peaks and their associated genes.

### 3.2 The TSWV genome contains m5C modifications

**Figure 2:**
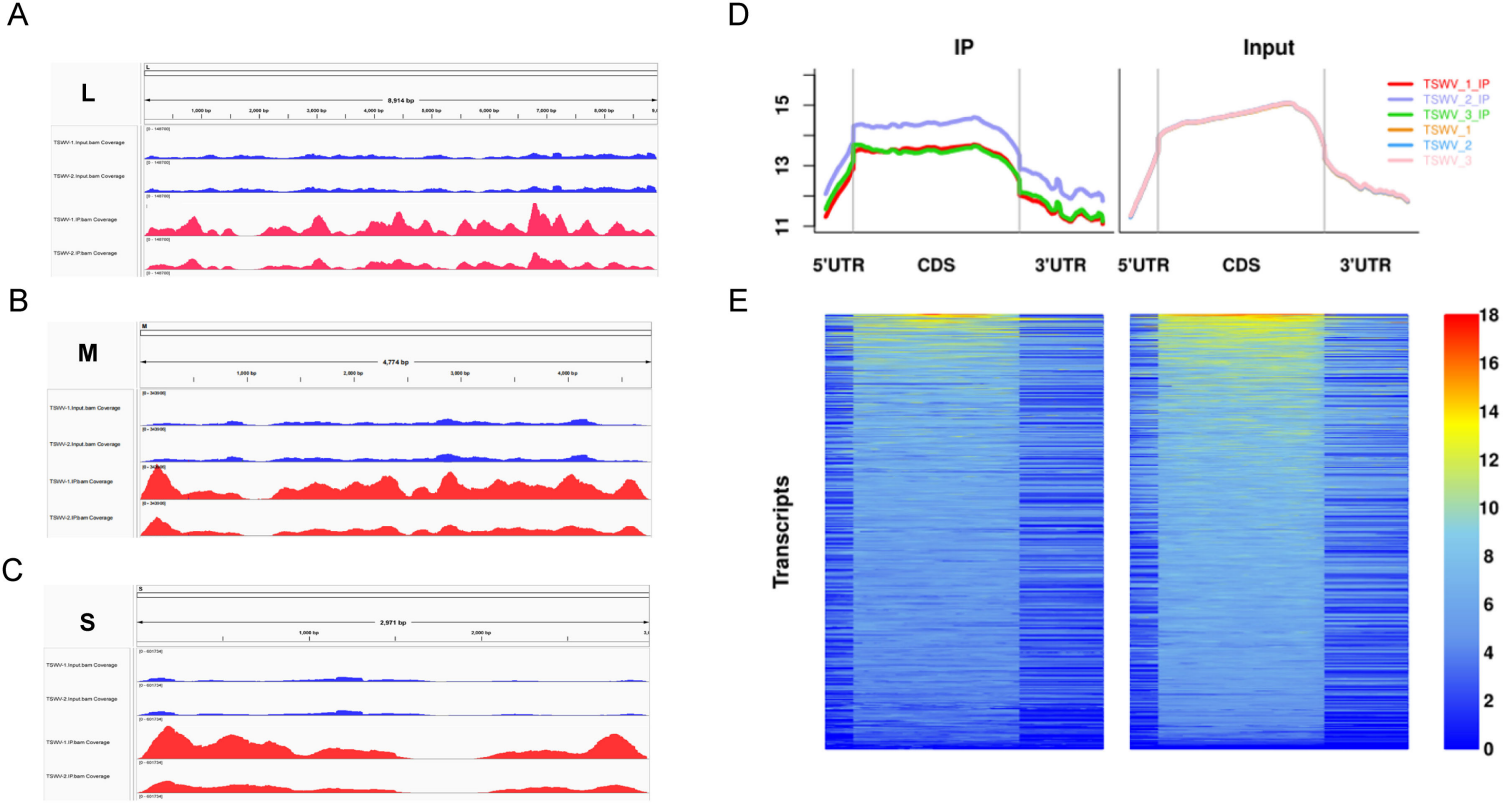
The TSWV genome contains m5C modifications. **(A)** TSWV-L 5-methylcytosine modification level. **(B)** TSWV-M 5-methylcytosine modification level. **(C)** TSWV-S 5-methylcytosine modification level. **(D)** Metagene profiles of m5C modification across TSWV transcripts. **(E)** Heatmap showing the distribution of m5C enrichment across TSWV transcripts.

Given that TSWV infection induces dynamic changes in m5C modifications of the tomato host transcriptome, we employed m5C-RIP-seq technology with two biological replicates each for the large (L), medium (M), and small (S) genomic RNA segments of Tomato spotted virus (TSWV)—the L segment encodes the viral RNA-dependent RNA polymerase, the M segment encodes movement proteins and glycoproteins, and the S segment encodes the nucleocapsid protein and a non-structural protein—to further investigate whether m5C modifications exist in the TSWV genome itself. By visualizing the m5C modification signals of the L, M, and S segments of the TSWV genome (**Figures 2A-C**), distinct m5C peaks were observed in all three segments, directly demonstrating the presence of m5C modifications across the entire TSWV genome. To further clarify the regional distribution pattern of m5C modifications throughout the TSWV genome, we performed an integrated analysis of the immunoprecipitation (IP, representing m5C enrichment) and input control signals from three samples (TSWV-1, TSWV-2, TSWV-3) (**Figure 2D**). The results showed that m5C enrichment signals were significantly higher in the CDS region than in the 5’-UTR and 3’-UTR. Heatmap analysis, which uses color intensity (corresponding to the degree of m5C modification enrichment) at the transcript level, directly shows that m5C modifications of TSWV are mainly concentrated in the CDS region, with their positional distribution primarily located after the start codon and before the stop codon (**Figure 2E**). In summary, reproducible m5C modifications exist in all three segments (L, M, and S) of the TSWV genome, and the m5C modifications across the viral genome exhibit a distribution preference biased toward the CDS region. This lays a foundation for subsequent elucidation of the regulatory role of m5C modifications in TSWV replication and pathogenic mechanisms.

### 3.3 Enrichment analysis of m5C-modified transcripts in tomato under TSWV infection

**Figure 3:**
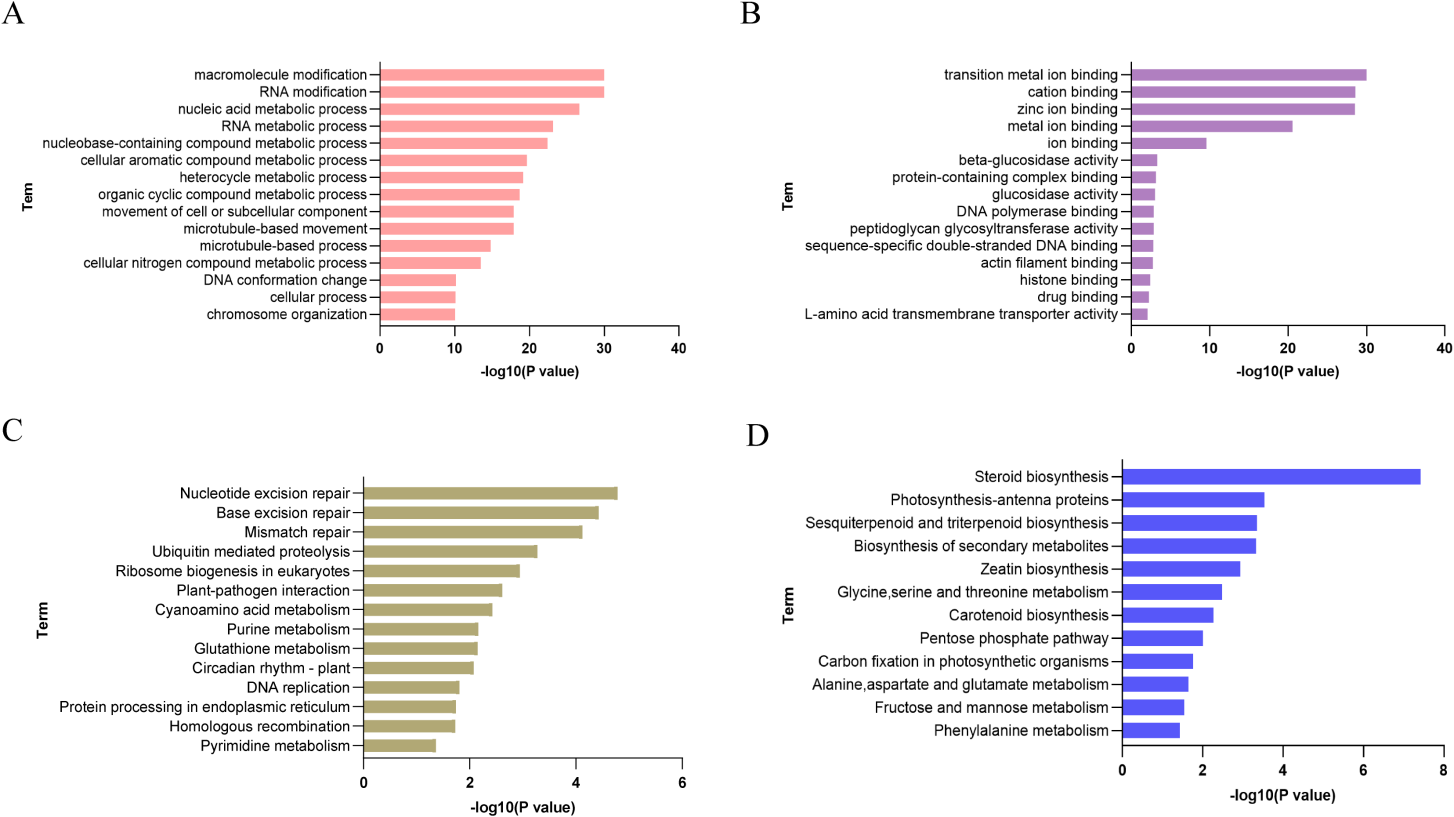
Enrichment analysis of m5C-modified transcripts in tomato with TSWV infection. **(A)** GO enrichment analysis of biological processes for upregulated m5C -modified transcripts in tomato. **(B)** GO enrichment analysis of molecular functions for upregulated m5C -modified transcripts in tomato. **(C)** KEGG enrichment analysis of upregulated m5C-modified transcripts in tomato. **(D)** KEGG enrichment analysis of downregulated m5C-modified transcripts in tomato.

To elucidate the potential biological functions of m5C methylation modification in tomato, we performed GO and KEGG pathway analyses on genes with m5C modification peaks, selecting upregulated and downregulated genes for analysis respectively. The biological process of GO enrichment analysis of upregulated genes showed that m5C methylated genes are involved in many key life activities, including macromolecule modification, RNA modification, nucleic acid-containing metabolic process, RNA metabolic process, and nucleobase-containing compound metabolic process (**Figure 3A**), indicating that m5C modification plays an indispensable role in RNA metabolism and gene expression regulation, basic cellular metabolism, and cellular structural organization and dynamic movement processes. Molecular function analysis showed that m5C methylated genes are enriched in transition metal ion binding, cation binding, zinc ion binding, and metal ion binding (**Figure 3B**), which directly links m5C methylation to various key biochemical and cellular activities such as substance binding, catalytic reactions, and molecular transport, reflecting its regulatory function at the molecular level. KEGG pathway analysis of upregulated genes found that m5C methylated genes are enriched in DNA repair (nucleotide excision repair, base excision repair, mismatch repair), protein degradation (ubiquitin-mediated proteolysis), ribosome biogenesis (eukaryotic ribosome biogenesis), plant-pathogen interaction, and various amino acid and nucleotide metabolism (cyanoamino acid metabolism, purine metabolism, pyrimidine metabolism) pathways (**Figure 3C**), emphasizing the importance of m5C methylation in the stability and transmission of genetic information, the guarantee of normal protein function, and metabolic balance and physiological regulation. KEGG pathway analysis of downregulated genes showed that m5C modification covers the biosynthesis and metabolism of various substances, including the synthesis of steroids, sesquiterpenes, triterpenes, secondary metabolites, etc., as well as the metabolism of glycine, serine, threonine, etc., and also involves key metabolic pathways such as photosynthesis related to photosynthetic antenna proteins, carbon fixation in photosynthetic organisms, and pentose phosphate pathway (**Figure 3D**), reflecting the important life activities of this modification in substance synthesis, energy transformation, and metabolic regulation. In general, these findings indicate that m5C methylation is a regulatory modification with diverse functions, widely involved in regulating various life processes of cells.

### 3.4 Host–virus integrated analysis of transcriptome and m5C methylome under TSWV infection

**Figure 4:**
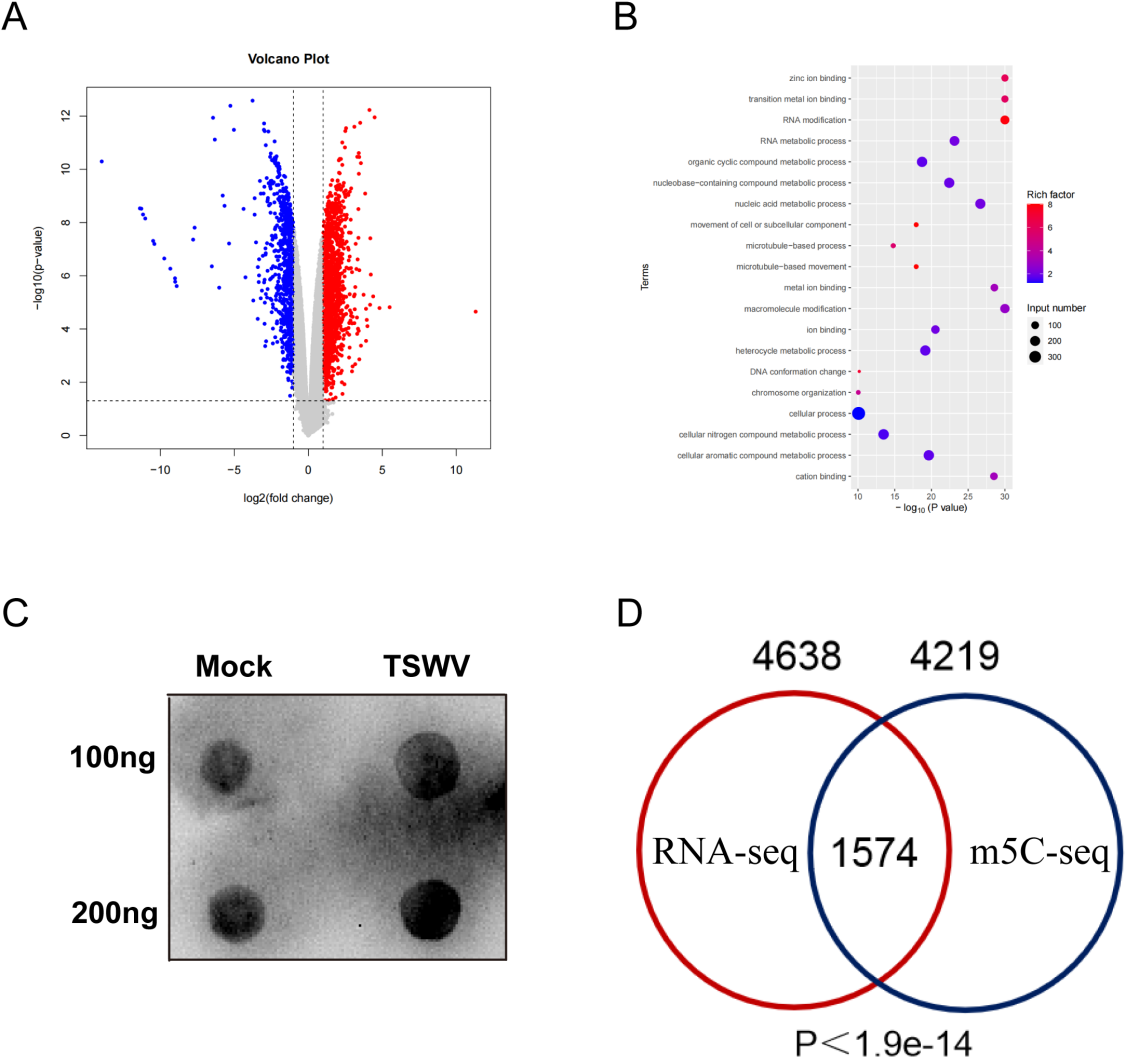
Integrated analysis of transcriptional and m5C modification dynamics under TSWV infection. **(A)** The volcano plot shows 2243 differentially expressed genes (DEGs) at the transcriptional level after TSWV infection, including 1388 upregulated genes (red) and 855 downregulated genes (blue). **(B)** RNA modifications enriched and RCMT family genes upregulated following TSWV infection. **(C)** Dot blot analysis validates the increase in global m5C levels upon TSWV infection. Total RNA from mock-inoculated (Mock) and TSWV-infected leaves was spotted in amounts of 100 ng and 200 ng per dot and probed with an anti-m5C antibody. The signal intensity was consistently stronger in the TSWV samples than in the Mock controls at both loading amounts, demonstrating that TSWV infection significantly enhances global RNA m5C methylation. (P < 0.001). **(D)** The Venn diagram shows the overlap of 1574 genes that are significantly altered in DMGs and DEGs.

To comprehensively characterize the host response to TSWV infection at both the transcriptional and epitranscriptomic levels, we first performed transcriptome-wide profiling to identify differentially expressed genes (DEGs). Comparative analysis revealed a total of 2,243 DEGs between TSWV-infected and mock-treated plants (**Figure 4A**), among which 1,388 genes were significantly upregulated (red) and 855 genes were significantly downregulated (blue). Intriguingly, gene set enrichment analysis demonstrated that RNA methyltransferases associated with m5C modification were broadly upregulated following viral infection (**Figure 4B**), suggesting a potential role of the m5C methylation machinery in the host antiviral response. Consistent with this observation, dot blot analysis revealed a marked increase in global RNA m5C modification levels in TSWV-infected samples compared to the mock controls (**Figure 4C**). To explore the interplay between transcriptional changes and m5C-mediated epitranscriptomic regulation, we integrated data from RNA-seq and m5C-seq. Venn diagram analysis identified 1,574 overlapping candidates that were both differentially expressed and differentially methylated (**Figure 4D**), a highly significant overlap (P < 1.9×10^−14^). This substantial convergence indicates a strong coordination between transcriptional and m5C-dependent post-transcriptional regulatory mechanisms during TSWV infection.

### 3.5 *SlTRM4B*-mediated m5C methylation positively regulates antiviral defense against TSWV

**Figure 5:**
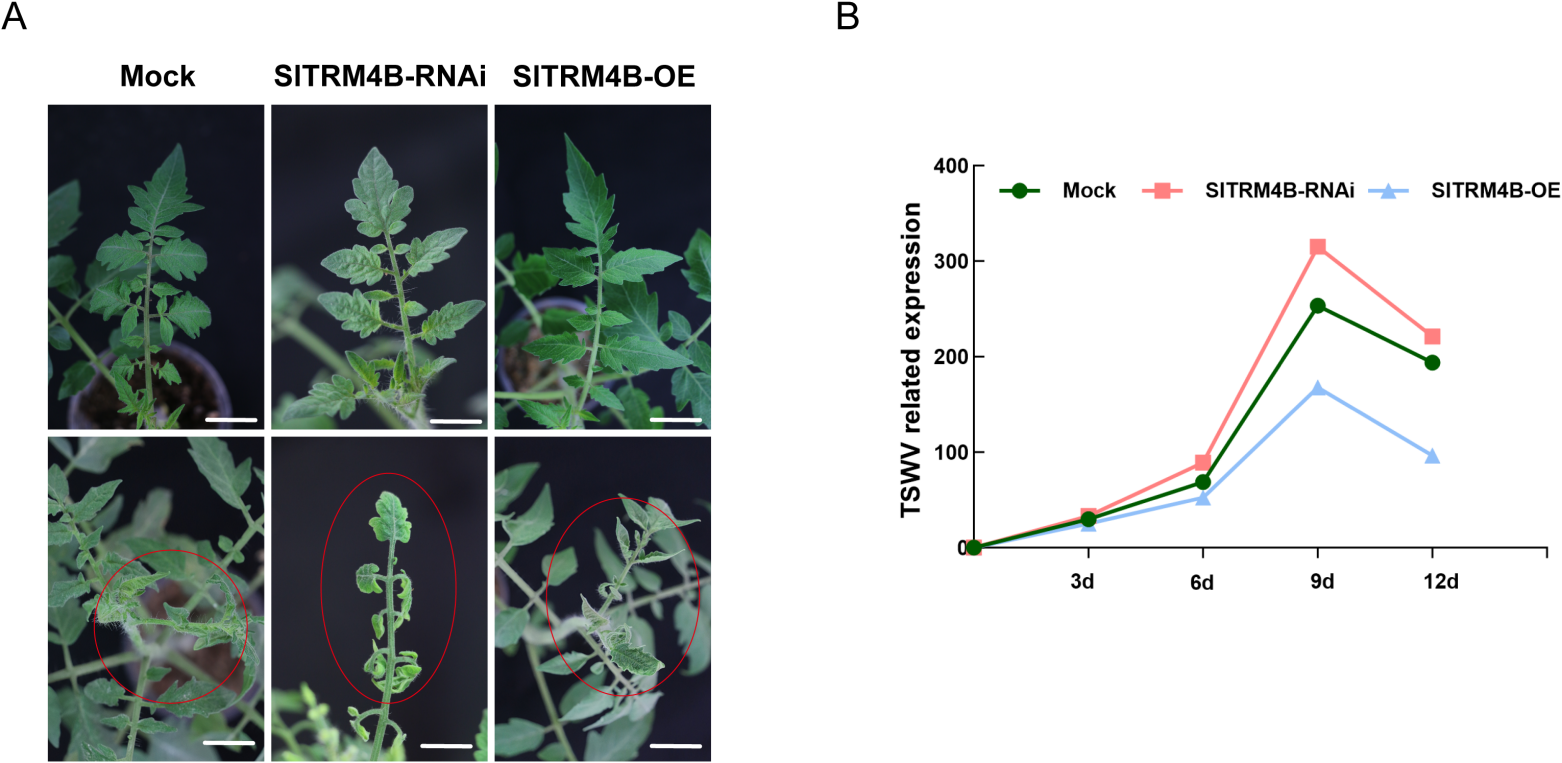
Silencing of *SlTRM4B* enhances susceptibility, whereas its overexpression confers resistance against TSWV. **(A)** Phenotypes of mock-inoculated (Mock), *SlTRM4B*-silenced (RNAi), and *SlTRM4B*-overexpressing (OE) tomato plants at 12 days post-inoculation (dpi) with TSWV. *SlTRM4B*-RNAi plants developed the most severe symptoms, including stunting and leaf necrosis, whereas *SlTRM4B*-OE plants displayed markedly attenuated symptoms. **(B)** Time-course analysis of relative TSWV accumulation levels determined by qRT−PCR. Data were normalized to an internal reference gene and are presented as mean ± SEM (n = 3). Viral accumulation was significantly higher in *SlTRM4B*-RNAi plants and significantly lower in *SlTRM4B*-OE plants compared with Mock controls across all time points (p < 0.05, two−way ANOVA), indicating that *SlTRM4B* expression negatively regulates viral replication.

We subjected mock-treated, *SlTRM4B*-OE, and *SlTRM4B*-RNAi tomato plants to TSWV infection, respectively. The results demonstrated that silencing of *SlTRM4B* led to severe disease symptoms, including leaf curling, necrosis, and growth retardation. In contrast, *SlTRM4B*-OE plants exhibited markedly milder symptoms, while mock-treated plants displayed an intermediate phenotype (**Figure 5A**). Time-course quantification of TSWV accumulation in the same set of plants was performed by qRT−PCR from 3 to 12 days post-inoculation (dpi). Viral RNA levels are presented as relative expression normalized to an internal control. *SlTRM4B*-RNAi plants supported the highest viral replication, showing a rapid increase and the highest peak titer at 9 dpi. Conversely, *SlTRM4B*-OE plants strongly suppressed viral accumulation, maintaining the lowest viral RNA levels throughout the infection period. Mock plants showed an intermediate viral load (**Figure 5B**). Taken together, the inverse correlation between *SlTRM4B* expression levels, disease severity, and viral load provides compelling genetic evidence that *SlTRM4B*-mediated m5C methylation functions as a positive regulator of antiviral defense against TSWV in tomato.

### 3.6 SlTRM4B is the putative mRNA m5C methyltransferase in tomato

**Figure 6:**
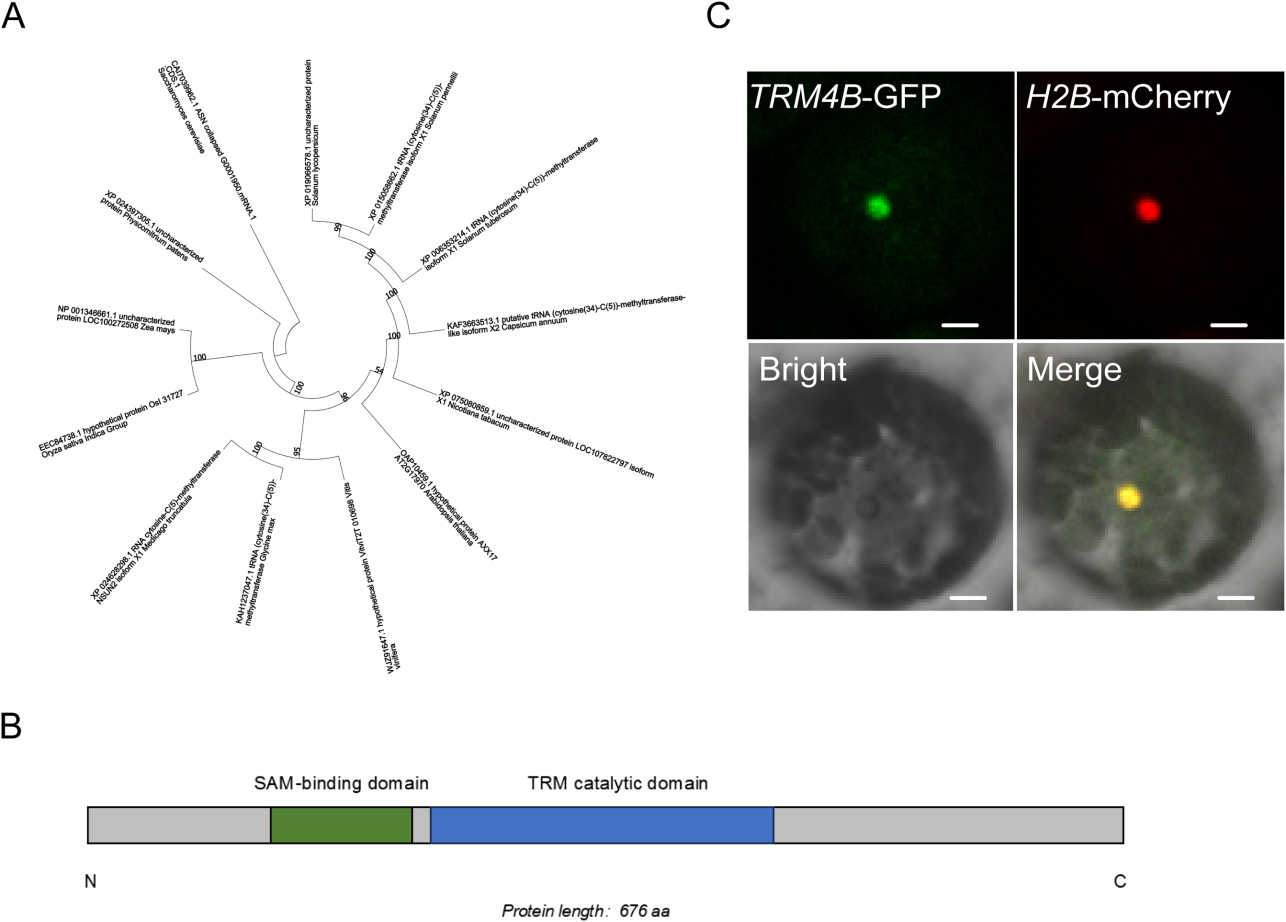
ntegrated analysis of tomato methyltransferase *TRM4B*. **(A)** Phylogenetic tree of the *TRM4B* family methyltransferases. **(B)** Conserved domain analysis of *TRM4B*. **(C)** Confocal microscopy analysis showed that the *TRM4B*-GFP fusion protein is localized in the nucleus of tomato protoplasts.

To assess whether SlTRM4B is a potential m5C methyltransferase in tomato, we first performed a phylogenetic analysis of TRM4B family proteins (Pavlopoulou and Kossida, 2009). The results demonstrated that SlTRM4B is highly homologous to functionally characterized m5C methyltransferases from other model plants and, notably, clusters in the same clade as AtTRM4B from Arabidopsis thaliana (**Figure 6A**), suggesting a conserved biological function. Subsequently, we predicted the conserved domains within the SlTRM4B protein sequence. This analysis revealed two canonical functional domains: an S-adenosylmethionine (SAM)-binding domain (amino acids 125-210) and a TRM catalytic domain (amino acids 220-440) (**Figure 6B**). The SAM-binding domain binds the methyl donor, whereas the TRM domain provides the catalytic core for cytosine-5 methylation. The presence of this domain architecture, which is highly consistent with known m5C “writer” enzymes like AtTRM4B, therefore provides strong evidence that SlTRM4B functions as an mRNA m5C methyltransferase in tomato. To validate the expression and localization of SlTRM4B in tomato, we transiently expressed a GFP-SlTRM4B fusion protein in tomato leaf protoplasts. Confocal microscopy revealed that the GFP signal was predominantly concentrated in the nucleus, where it co-localized with the nuclear marker H2B-mCherry (**Figure 6C**). This nuclear localization is consistent with a role in post-transcriptional mRNA modification.

### 3.7 *SlTRM4B* regulates mRNA stability under TSWV infection stress in tomato

**Figure 7:**
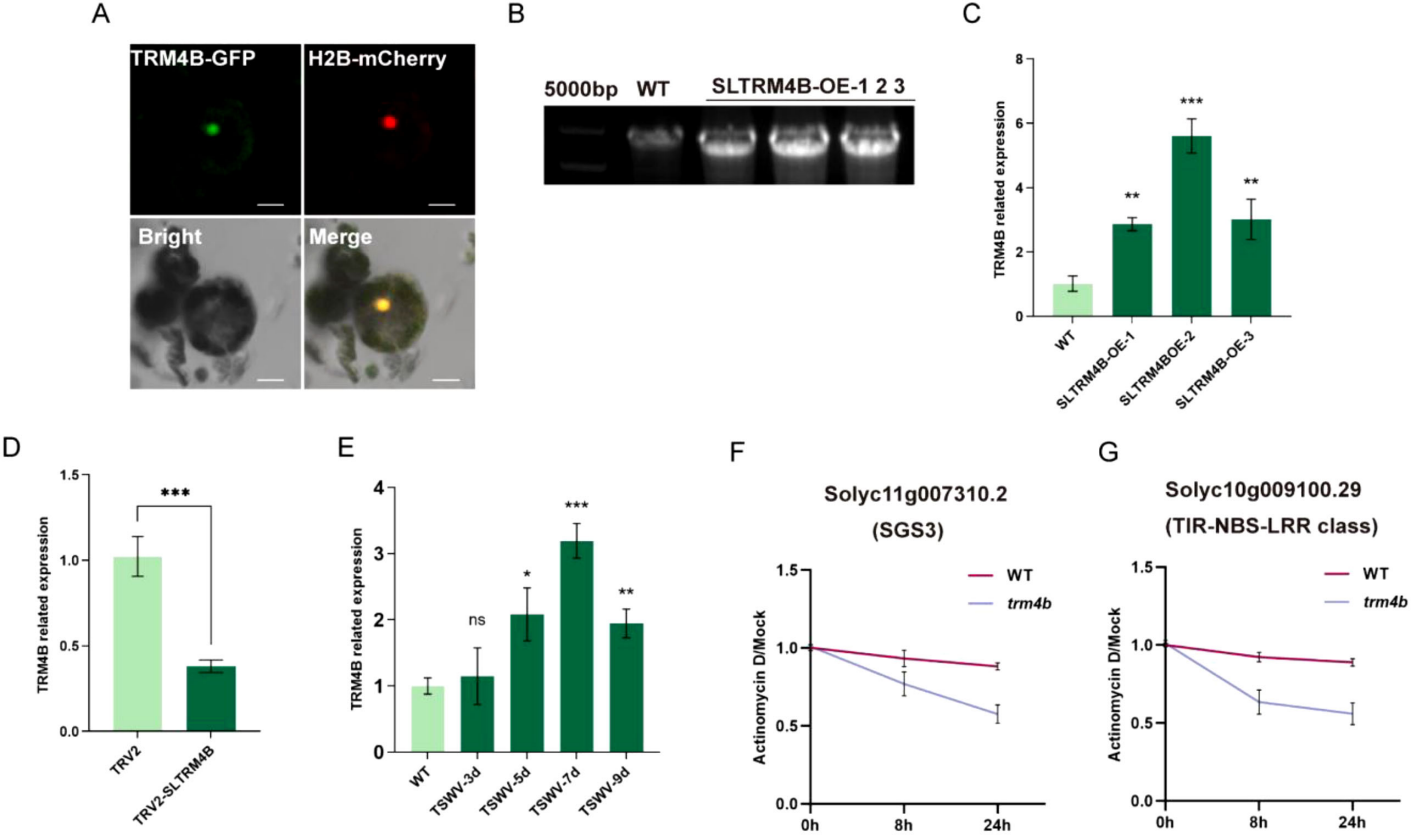
Functional characterization of *TRM4B* in response to TSWV infection. **(A)** Confocal microscopy analysis revealed nuclear localization of *SlTRM4B*-GFP in protoplasts of *SlTRM4B*-OE plants. **(B)** RT-PCR validation confirmed successful transcription of the *SlTRM4B* gene in *SlTRM4B*-OE plants. **(C)** Relative expression levels of *SlTRM4B* in WT and three independent *SlTRM4B*-OE lines, with all transgenic lines showing significantly higher *SlTRM4B* expression than WT. **(D)** Relative expression levels of SlTRM4B in silenced plants, with the empty vector (TRV2) used as the control. Statistical analysis was performed using one-way analysis of variance (ANOVA) (* p < 0.05, ** p < 0.01, *** p < 0.001). Data are means ± standard deviation (SD) (n = 3 biological replicates). **(E)** Relative expression levels of *SlTRM4B* in tomato leaves at days 3, 5, 7, and 9 after TSWV treatment. **(F, G)** Actinomycin D treatment of *trm4b* knockout plants at 0, 8, and 24 hours demonstrated the role of *SlTRM4B* in regulating tomato transcript stability under TSWV infection conditions.

To investigate the role of *SlTRM4B* during TSWV infection, we first generated and validated both *SlTRM4B*-overexpressing (OE) and silenced plant lines. RT-PCR and qPCR analyses confirmed significantly increased *SlTRM4B* transcript levels in three independent OE lines compared to WT (**Figure 7B, C**), and markedly reduced expression in TRV2-mediated silenced plants (**Figure 7D**). We found that *SlTRM4B* expression was significantly induced in tomato leaves following TSWV infection (**Figure 7E**), suggesting its involvement in the viral response. To test whether *SlTRM4B* affects RNA stability in tomato, we treated plants with actinomycin D to inhibit new RNA synthesis. In *trm4b* mutants, the stability of RNA was substantially lower at 8 and 24 hours compared to WT plants (**Figures 7F, G**). This result indicates that *SlTRM4B* contributes to maintaining RNA stability during viral infection. This function is supported by the nuclear localization of GFP-tagged *SlTRM4B* in OE leaf protoplasts (**Figure 7A**), which is consistent with a role in nuclear RNA metabolism. Collectively, these results demonstrate that *SlTRM4B* is induced by TSWV and plays a critical role in enhancing the stability of specific mRNAs under viral stress.

## 4 Discussion

The dynamic and reversible nature of epitranscriptomic modifications, regulated by writer proteins, eraser proteins, and reader proteins, adds an additional regulatory layer to gene expression (Motorin and Grosjean, 1999; Wang et al., 2023). Although m5C was identified decades ago, its functional characterization in plants, especially in horticultural crops under biotic stress, has progressed more slowly than that of m6A (Roundtree et al., 2017). In this study, we constructed a transcriptome wide map of m5C in tomato and found that methylation peaks are broadly distributed across chromosomes. The modifications show clear positional preferences that include enrichment near translation start sites and within coding sequences, together with conserved sequence features that are consistent with reports in *Arabidopsis* and rice (Cui et al., 2017; Tang et al., 2020). These shared patterns support the view that m5C is an evolutionarily conserved regulatory mechanism in plants and may contribute to the control of translation and transcript stability.

A major finding of this work is that TSWV infection triggers a global increase in m5C levels in tomato leaves. The transcripts showing increased methylation were strongly enriched for immune and stress related pathways, including plant pathogen interaction. Notably, hypermethylation was positively associated with increased transcript abundance, which is consistent with the idea that m5C can enhance mRNA stability and support effective gene expression during stress (Wang et al., 2023). In parallel, multiple members of the RNA cytosine methyltransferase related gene set were induced, with *SlTRM4B* showing a prominent response. Functional assays further demonstrated that *SlTRM4B* contributes to antiviral defense. Silencing *SlTRM4B* resulted in enhanced disease severity and increased viral accumulation, while overexpression conferred reduced symptoms and lower viral loads. Moreover, inhibition of transcription revealed that loss of *SlTRM4B* reduced the stability of selected target transcripts under infection conditions. Together, these results support a model in which TSWV infection induces *SlTRM4B*, which in turn increases m5C deposition on defense related mRNAs, thereby promoting their stability and supporting a stronger antiviral response.

Intriguingly, our m5C RIP seq data also revealed the presence of m5C modifications on the TSWV genomic RNA segments themselves. These modifications were preferentially enriched in the CDS regions. This observation raises compelling questions regarding host virus interactions. In some viruses, m5C modifications can promote viral RNA stability or modulate innate immune sensing (Feng et al., 2023; Wang et al., 2024). However, in the context of Drosophila C virus, the host RNA m5C methyltransferase Dnmt2 contributes to antiviral defense (Chen et al., 2021). Given that *SlTRM4B* overexpression in our study suppressed TSWV accumulation, the m5C deposition on viral RNA could potentially reflect a host imposed restriction mechanism that marks viral RNA for degradation or translational inhibition. Alternatively, it may represent competition in which the virus attempts to utilize host methylation machinery for its own benefit, while the host simultaneously elevates methylation on defense transcripts to tip the balance toward resistance. Determining whether m5C on TSWV RNA benefits the virus or the host will require future functional dissection of viral m5C sites.

While we have identified *SlTRM4B* as a key m5C writer, the complete regulatory circuit remains to be elucidated. The specific reader proteins that recognize these m5C marks in tomato and execute downstream outcomes are unknown. Furthermore, the nuclear localization of *SlTRM4B* suggests that methylation may occur during transcription, but how modified transcripts are selected for export and stabilized in the cytoplasm under stress still needs clarification. Future research combining spatial transcriptomics with RNA protein interaction profiling will be essential to map the spatiotemporal dynamics of TRM4B and identify its downstream effectors.

In conclusion, this study provides a comprehensive analysis of dynamic changes in the tomato m5C methylome under viral infection and functionally characterizes *SlTRM4B* as a key mRNA m5C methyltransferase in this process. We demonstrate that *SlTRM4B* mediated m5C hypermethylation is not merely a passive consequence of infection but an active defense strategy that enhances the stability of stress responsive transcripts. These findings highlight the potential of manipulating RNA methylation pathways as a promising avenue for breeding virus resistant crops.

## Data Availability

The datasets presented in this study can be found in online repositories. The names of the repository/repositories and accession number(s) can be found in the article/**Supplementary Material**.
